# Calcium Intake and the Risk of Ovarian Cancer: A Meta-Analysis

**DOI:** 10.3390/nu9070679

**Published:** 2017-06-30

**Authors:** Xingxing Song, Zongyao Li, Xinqiang Ji, Dongfeng Zhang

**Affiliations:** 1 Department of Epidemiology and Health Statistics, the College of Public Health of Qingdao University, 38 Dengzhou Road, Qingdao 266021, Shandong, China; songxx1217@163.com (X.S.); lizy199002@163.com (Z.L.); 2 Modern Educational Technology Center, Qingdao University, Qingdao 266021, Shandong, China; jixinqiang@qdu.edu.cn

**Keywords:** calcium, intake, ovarian cancer, meta-analysis

## Abstract

Several epidemiological studies have evaluated the association between calcium intake and the risk of ovarian cancer. However, the results of these studies remain controversial. Thus, we performed a meta-analysis to explore the association between calcium intake and the risk of ovarian cancer. Pubmed, Embase and Web of Science were searched for eligible publications up to April 2017. Pooled relative risks (RRs) with 95% confidence intervals (CIs) were calculated using the random-effects model. Small-study effect was estimated using Egger’s test and the funnel plot. Among 15 epidemiological studies involving 493,415 participants and 7453 cases eligible for this meta-analysis, 13 studies were about dietary calcium intake, 4 studies about dairy calcium intake and 7 studies about dietary plus supplemental calcium intake. When comparing the highest with the lowest intake, the pooled RRs of ovarian cancer were 0.80 (95% CI 0.72–0.89) for dietary calcium, 0.80 (95% CI 0.66–0.98) for dairy calcium and 0.90 (95% CI 0.65–1.24) for dietary plus supplemental calcium, respectively. Dietary calcium was significantly associated with a reduced risk of ovarian cancer among cohort studies (RR = 0.86, 95% CI 0.74–0.99) and among case-control studies (*RR* = 0.75, 95% CI 0.64–0.89). In subgroup analysis by ovarian cancer subtypes, we found a statistically significant association between the dietary calcium (*RR* = 0.78, 95% CI 0.69–0.88) and the risk of epithelial ovarian cancer (EOC). This meta-analysis indicated that increased calcium intake might be inversely associated with the risk of ovarian cancer; this still needs to be confirmed by larger prospective cohort studies.

## 1. Introduction

Ovarian cancers include three major histologic types: epithelial, sex cord/stromal and germ cell cancer. Approximately 90% of ovarian cancers are classified as epithelial ovarian cancer (EOC) [1,2]. There are more than 200,000 new ovarian cancer cases and 140,000 deaths of ovarian cancer per year, globally [3]. Ovarian cancer is the seventh most common cause of cancer death among women worldwide and the fifth leading cause of cancer death among women in the United States (US) [3,4].

The majority of cases are usually diagnosed at an advanced stage [5,6], contributing to poor survival. Given the suboptimal prognosis for this disease [7], it is necessary to explore modifiable risk factors to prevent ovarian cancer. Several factors have been confirmed to be associated with the risk of ovarian cancer, such as inheritance [8], anthropometric factors [9], hormonal and reproductive factors [10]. For dietary and nutritional factors, no specific dietary factors are consistently implicated in ovarian cancer [11]. Nonetheless, several meta-analyses have found that dairy products [12] and egg [13] may increase the risk of ovarian cancer, while fish [14], soy [15] and vegetables [16] may reduce the risk of ovarian cancer.

As an important component in foods, calcium has been identified as being associated with many diseases, for instance, cardiovascular disease [17], stroke [18] and breast cancer [19]. Some studies have found that calcium intake may play a role in the development of ovarian cancer [20,21,22]. Accordingly, numerous epidemiological studies have been carried out to evaluate the association between calcium intake and the risk of ovarian cancer. However, the results are inconsistent [23,24,25,26,27,28,29,30,31,32,33,34,35]. Four studies found that calcium intake was inversely related to ovarian cancer risk [27,30,31,32], while other studies found no evidence of an association [23,24,25,26,28,29,33,34,35]. Therefore, we systematically conducted a meta-analysis to (1) further investigate the associations between dietary calcium and dairy calcium intake and the risk of ovarian cancer; (2) further explore the effect of dietary plus supplemental calcium intake on the risk of ovarian cancer.

## 2. Materials and Methods

We followed the Preferred Reporting Items for Systematic reviews and Meta-Analyses (PRISMA) guidelines in this meta-analysis [36].

### 2.1. Literature Search Strategy

A literature search was performed up to April 2017 for relevant available articles from the PubMed, Embase and Web of Science databases. We used the search terms “nutrition” OR “diet” OR “dietary” OR “calcium” in combination with (“ovarian” OR “ovary”) and (“neoplasm” OR “carcinoma” OR “cancer” OR “tumor”). We also reviewed the reference lists of the included studies for undetected relevant studies.

### 2.2. Inclusion Criteria

The inclusion criteria were as follows: (1) a case-control or cohort study published as an original study; (2) the exposure of interest was calcium intake; (3) the outcome of interest was ovarian cancer; (4) relative risk (RR) with 95% confidence interval (CI) were available (or data could be calculated). (5) the most recent and complete study was selected if data from the same population had been published more than once.

All studies were carefully searched and reviewed independently by two investigators. If the two investigators disagreed about the eligibility of an article, it was resolved by consensus with a third reviewer.

### 2.3. Quality Assessment

The Newcastle-Ottawa quality assessment scale was used to assess the quality of the original studies. Quality of selection, comparability, and exposure or outcome of study participants are three major parameters. And a higher score represents better methodological quality.

### 2.4. Data Extraction

The following data were extracted from each study by two investigators independently: the first author, publication year, country in which the study was conducted, study design, follow-up duration, age range or mean age at baseline, sample size, number of cases, dietary assessment method, the most adjusted RR with 95% CI for the highest versus lowest category of the intake of calcium, and the covariates that were adjusted for in each study.

### 2.5. Statistical Analysis

Pooled measure was calculated as the inverse variance-weighted mean of the logarithm of RR with 95% CI to assess the strength of association between calcium intake and the risk of ovarian cancer. The random effect model was used to combine study-specific RRs (95% CIs) [37]. The *I*^2^ was adopted to assess the heterogeneity between studies (*I*^2^ values of 0%, 25%, 50% and 75% represented no, low, moderate and high heterogeneity, respectively) [38]. Meta-regression was performed to assess the potentially important covariates that might exert substantial impacts on between-study heterogeneity. We also conducted subgroup analyses stratified by study design, continent, whether the study adopted validated food frequency questionnaires (FFQs) as the exposure assessment method, and whether the results were adjusted for covariates of parity and tubal ligation. Influence analysis was performed with one study removed at a time to assess whether the results could have been affected markedly by a single study [39]. In the cumulative meta-analysis, studies were added one at a time according to the published year, and the results were summarized sequentially. Small-study effect was assessed with visual inspection of the funnel plot and Egger’s test [40].

All statistical analyses were performed with STATA version 12.0 (Stata Corporation, College Station, TX, USA). All reported probabilities (*p*-values) were two-sided with *p* ≤ 0.05 considered statistically significant.

## 3. Results

### 3.1. Literature Search and Study Characteristics

**Figure 1:**
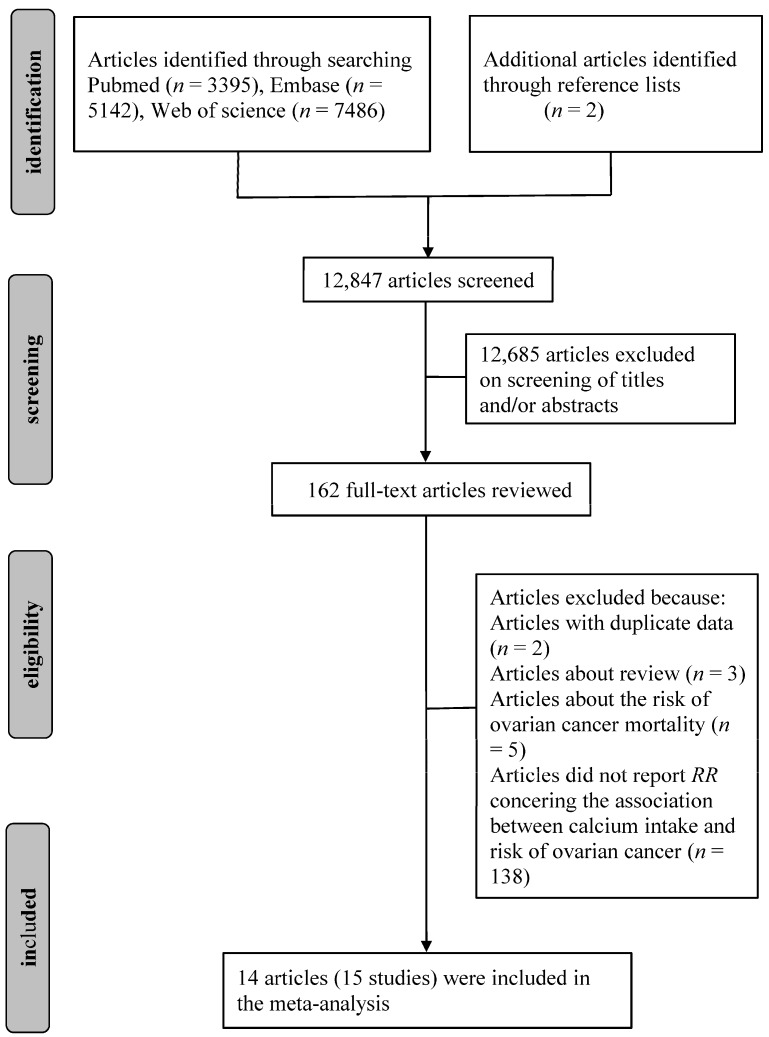
Flowchart of the selection of studies included in the meta-analysis.

Initially, 3395 articles from Pubmed, 7486 from Web of Science and 5142 from Embase were identified. Two additional articles were also found from reference lists. After reviewing the titles and abstracts, 162 articles about the association of calcium intake with risk of ovarian cancer were identified. After reviewing the full texts, 148 articles were subsequently excluded: two were from the same population; three were systematic review; five were about the risk of ovarian cancer mortality and 138 did not provide RR concerning the association between calcium intake and the risk of ovarian cancer. Finally, a total of 14 published articles [23,24,25,26,27,29,30,31,32,33,34,35,41,42], including 15 studies were eligible for this meta-analysis. The detailed steps of our literature search are shown in Figure 1.

Among these included studies, 13 studies evaluated the relationship between dietary calcium and risk of ovarian cancer [24,25,26,27,29,30,31,32,33,34,35,42]. Seven studies evaluated the relationship between dietary plus supplemental calcium and risk of ovarian cancer [23,25,27,30,35,41,42]. Four studies evaluated the relationship between dairy calcium and risk of ovarian cancer [26,29,32]. With regard to the location, 11 studies were conducted in North America [23,25,27,29,30,32,34,35,41,42] and four studies in Europe [24,26,31,33]. As for study design, eight studies were case-control studies [24,26,27,30,31,32,33,34], and seven were cohort studies [23,25,29,35,41,42]. 11 studies used validated food frequency questionnaires (FFQs) for the assessment of calcium intake [23,25,27,29,30,31,32,34,35,42]. The basic characteristics of the included studies for calcium intake with risk of ovarian cancer are shown in Table 1. The quality assessment showed that the Newcastle-Ottawa score of each study was not less than 7, indicating that the methodological quality was generally good. The quality assessment result is showed in Supplementary Material Table S1.

### 3.2. Quantitative Synthesis

The main results are summarized in Table 2.

#### 3.2.1. Dietary Calcium and the Risk of Ovarian Cancer

**Figure 2:**
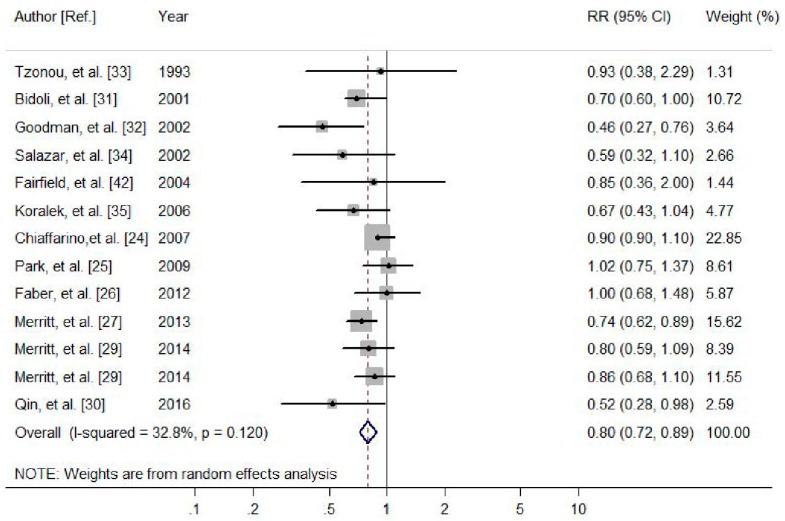
Meta-analysis of the association between dietary calcium intake and ovarian cancer risk. The size of the gray box is positively proportional to the weight assigned to each study, which is inversely proportional to the standard error of the relative risks, and horizontal lines represent the 95% confidence intervals.

A total of 13 studies, with eight case-control studies and five cohort studies were included, involving 367,057 participants and 7034 cases. Four studies revealed a significant association between dietary calcium intake and the risk of ovarian cancer, while the other nine studies found no association. For the highest vs. lowest category of dietary calcium intake, the pooled RR of ovarian cancer was 0.80 (95% CI 0.72–0.89, *I*^2^ = 32.8%, *P*_heterogeneity_ = 0.120, Figure 2). In further analysis by ovarian cancer subtypes, dietary calcium intake was also significantly associated with a reduced risk of EOC (RR = 0.78, 95% CI: 0.69–0.88).

In subgroup analysis stratified by continent in which the studies were conducted, dietary calcium was significantly associated with decreased ovarian cancer risk among studies conducted in North America (*RR* = 0.76, 95% CI 0.66–0.87) and Europe (RR = 0.86, 95% CI 0.75–0.99). When stratified by study design subtype, a statistically significant effect of dietary calcium on ovarian cancer risk was observed both among case-control studies (RR = 0.75, 95% CI 0.64–0.89) and cohort studies (RR = 0.86, 95% CI 0.74–0.99). For subgroup analysis stratified by dietary assessment method, the inverse association was also statistically significant in validated FFQs group (RR = 0.75, 95% CI 0.67–0.85) and in no-validated FFQs group (RR = 0.91, 95% CI 0.82–1.00). The remaining results of subgroup analyses are shown in Table 2.

#### 3.2.2. Dietary Plus Supplemental Calcium Intake and the Risk of Ovarian Cancer

For dietary plus supplemental calcium intake, seven studies (two case-control studies and five cohort studies) involving 317,995 participants and 3780 cases were included. Two studies revealed a significant association between dietary plus supplemental calcium intake and the risk of ovarian cancer, while the other five studies found no association. The pooled RR of ovarian cancer was 0.90 (95% CI 0.65–1.24, *I*^2^ = 76.1%, *P*_heterogeneity_ = 0.0001, Supplementary Material Figure S1) for the highest vs. lowest category of dietary plus supplemental calcium intake.

#### 3.2.3. Dairy Calcium Intake and the Risk of Ovarian Cancer

For dairy calcium intake, four studies (two case-control studies and two cohort studies) involving 155,859 participants and 2330 cases were included, and the pooled RR of ovarian cancer was 0.80 (95% CI 0.66–0.98, *I*^2^ = 34.5%, *P*_heterogeneity_ = 0.205, Supplementary Material Figure S2) for the highest vs. lowest category of dairy calcium intake.

### 3.3. Cumulative Meta-Analysis

**Figure 3:**
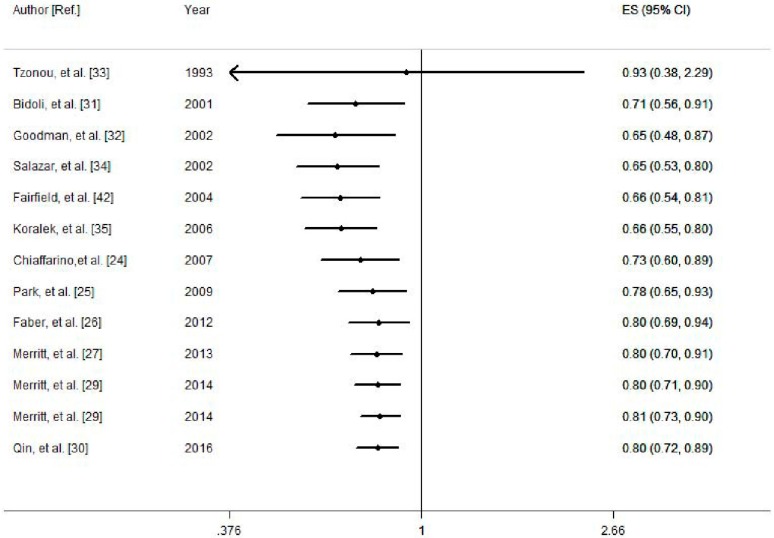
Cumulative meta-analysis of the association between dietary calcium intake and ovarian cancer risk. Open circle indicates the pooled relative risks, horizontal line represents the 95% confidence intervals.

Cumulative meta-analysis for the association between dietary calcium and the risk of ovarian cancer was conducted to indicate the dynamic trend of results and assess the influence of an individual study on the overall results (Figure 3). The results indicated that there was not an association between dietary calcium and the risk of ovarian cancer until adding the study conducted in 2001 [31] (cumulative RR: 0.71 (95% CI: 0.56–0.91)). Since 2012, the significant association remained stable thereafter (cumulative RR: 0.80 (95% CI: 0.69–0.94)).

### 3.4. Meta-Regression and Influence Analysis

Univariate meta-regression with covariates was conducted to explore the source of heterogeneity. In analysis of dietary calcium with risk of ovarian cancer, we performed univariate meta-regression with the covariates of sample size (*p* = 0.162), continent (*p* = 0.296), study design (*p* = 0.394), whether adjusted for energy intake (*p* = 0.319), parity (*p* = 0.584), oral contraceptive use (*p* = 0.896), tubal ligation (*p* = 0.196) and dietary assessment method for calcium intake (*p* = 0.033). The results showed that the dietary assessment method contributed to the between-study heterogeneity.

In analysis of dietary plus supplemental calcium with risk of ovarian cancer, we performed univariate meta-regression with the covariates of sample size (*p* = 0.150), study design (*p* = 0.029), dietary assessment method (*p* = 0.190) and whether adjusted for parity (*p* = 0.874), oral contraceptive use (*p* = 0.190) and tubal ligation (*p* = 0.401). The results showed that the study design contributed to the between-study heterogeneity.

In an influence analysis excluding one study at a time, no individual study had an excessive influence on the above-mentioned pooled effects (Supplementary Material Figures S3 and S4).

### 3.5. Small-Study Effect Evaluation

**Figure 4:**
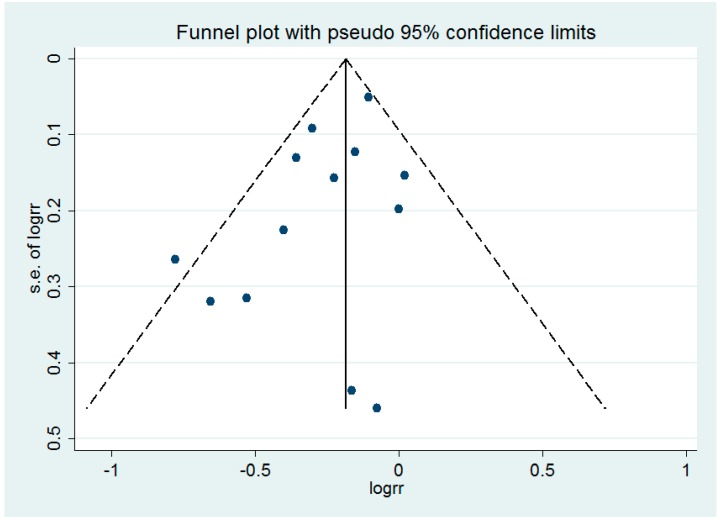
Funnel plot for the analysis of dietary calcium intake and ovarian cancer risk. Each dot represents a different study.

Egger’s test showed no evidence of a significant small-study effect for the analyses between the consumption of dietary calcium (*p* = 0.095), dietary plus supplemental calcium (*p* = 0.501) and the risk of ovarian cancer. The funnel plot of the analysis of dietary calcium and the risk of ovarian cancer is shown in the Figure 4.

## 4. Discussion

This meta-analysis evaluated associations between the intake of dietary calcium, dietary plus supplemental calcium and dairy calcium and the risk of ovarian cancer, respectively. A total of 15 studies with 493,415 participants and 7453 cases were included. The results indicated that there were inverse associations between the intake of dietary calcium, dairy calcium and the risk of ovarian cancer, respectively. In subgroup analyses, there was a significant inverse association between dietary calcium and the risk of ovarian cancer for studies carried out in North America and Europe. The association was also significant among results in cohort studies and case-control studies. In cumulative meta-analysis, the above-mentioned significant association was first observed after adding one study in 2001, and the result tended to be stable since 2012. However, a pooled analysis of 12 cohort studies in 2006 indicated that intake of dairy calcium was not associated with ovarian cancer risk. In contrast to our study, such inconsistencies may be due in part to the different sources of calcium intake. The sources of dietary calcium are not only dairy products but also other sources including shrimp, broccoli, leafy green vegetables, etc. In addition, the absorption of calcium from the dairy and other diet is affected by different factors. The association of dietary plus supplemental calcium with ovarian cancer risk was not statistically significant. Presumably, the dose of dietary plus supplemental calcium was relatively high and different in the control group across studies, which could influence the effect of the result. Moreover, the number of studies for dietary plus supplemental calcium was relatively small and this could also affect the final result.

The relatively insufficient sample size and dietary measurement error of individual study could be likely to contribute to the inconsistency, thus we conducted the present meta-analysis to increase the sample size and improve the study power. In addition, considering the recall and select bias of case-control study, we conducted subgroup analysis stratified by the study design, but the results were not substantially affected.

The exact biological mechanisms underlying calcium intake and risk of ovarian cancer are still not completely determined. One underlying explanation for our findings is that a higher level of calcium might be inversely related to ovarian cancer risk via down-regulation of circulating parathyroid hormone (PTH) [21,22]. The reduction of PTH could decrease hepatic and osteoblastic synthesis of insulin-like growth factor-1 (IGF-1) [22,43]. IGF-1 may exert a direct effect by increasing cell proliferation and inhibition of apoptosis [44], and experimental studies have indeed shown that malignant transformation of ovarian epithelial cells (the cells from which ovarian cancer is believed to originate) can be induced by overexpression of the IGF-1 receptor [45]. These mitogenic and anti-apoptotic effects of IGF-I might be particularly relevant during ovulation related tissue remodeling of the surface epithelium [46]. The reduction of IGF-1 would weaken mitogenic effects on the pathogenesis of ovarian cancer [47,48]. In addition, PTH may be a tumour promoter acting as a co-mitogen and anti-apoptotic factor directly [22,49].

Between-study heterogeneity is common in meta-analysis [50], and it is essential to explore the potential sources of between-study heterogeneity. Diversity in population stratification, measurement of calcium intake and variation of the covariates might be the source of between-study heterogeneity. High between-study heterogeneity was found in the analysis of dietary plus supplemental calcium and risk of ovarian cancer. In meta-regression, we found that the study design contributed to between-study heterogeneity. In subgroup analysis by study design, the heterogeneity decreased to 44.8% (*P*_heterogeneity_ = 0.124) for cohort studies and 0% (*P*_heterogeneity_ = 0.508) for case-control studies, respectively. Additionally, low between-study heterogeneity was found in the analysis of dietary calcium and risk of ovarian cancer. In meta-regression, we found that the dietary assessment method was the contributor to between-study heterogeneity. In subgroup analysis by studies adjusted for dietary assessment method or not, the heterogeneity decreased to 20.5% (*P*_heterogeneity_ = 0.254) and 0% (*P*_heterogeneity_ = 0.875), respectively. The results were also consistent with the overall pooled RR, indicating that our results were stable and reliable.

There are several strengths in present meta-analysis. First of all, this study included a large number of participants and cases, allowing a much great possibility of reaching reasonable conclusions. Second, most of the studies had adjusted for potential confounders, such as age, energy and oral contraceptives, strengthening the credibility of the results. Third, most of the included studies used validated FFQs, ensuring the accuracy of dietary assessment. Fourth, a statistically significant inverse association between dietary calcium and ovarian cancer risk was found in cohort studies, indicating a potential causal relationship between them. Fifth, in cumulative meta-analysis by publication year, the significant result persisted and the CI became increasingly narrower.

However, several limitations of the study should also be considered. First, potential confounders adjusted for in each study were different and it might affect the results to some extent. For example, parity and tubal ligation were adjusted for in some studies, while they not adjusted for in other studies. In addition, residual confounding should be of concern. Second, most studies just used a complete dietary assessment, and it could not reflect change in diet for a long time. Third, the standards of lowest calcium intake were inconsistent among studies, which might influence the result. Fourth, case-control studies, which are prone to recall and select biases, were included in our meta-analysis, and the results would be partly affected. Fifth, the number of included studies for dairy calcium was too small (only four studies). Although the result of dairy calcium with risk of ovarian cancer was statistically significant, it was different from the result reported by Genkinger et al. in 2006 [28]. Sixth, we were unable to explore the dose–response relationship between the intake of calcium and the risk of ovarian cancer because of the limited data.

## 5. Conclusions

In summary, this meta-analysis revealed that dietary calcium and dairy calcium, but not dietary plus supplemental calcium, may reduce the risk of ovarian cancer. Increasing intake of dietary calcium should be advocated for the primary prevention of ovarian cancer. Better designed prospective cohort studies are needed to explore the association between calcium intake and ovarian cancer risk.

## Figures and Tables

**Table 1:** Characteristics of the studies included on the intake of calcium and the risk of ovarian cancer.

Author [Ref.]	Year	Country	Age Range/Mean Age (Case/Control)	Follow Years (Median)	Study Design	Dietary Assessment	Sample Size (Case)	Range of Calcium (Highest/Lowest) (mg/Day)	Exposure	Outcome	RR (95% CI)	Adjustment for Covariates
Goodman, M.T. [32]	2002	US	54.8	NA	CC	Validated FFQ	1165 (558)	Highest: >1107.9 Lowest: <528.1	Dietary calcium	EOC	0.46 (0.27, 0.76)	Age, ethnicity, study center, education, energy intake, parity, oral contraceptive use, tubal ligation
								Highest: >631.4 Lowest: <182.9	Dairy calcium	EOC	0.55 (0.36, 0.84)	
Merritt, M.A. [29]	2014	US	25–55	28	Cohort	Validated FFQ	76243 (609)	Highest: >1018 Lowest: <433	Dairy calcium	EOC	0.80 (0.59, 1.09)	Total caloric intake, menopausal status, number of pregnancies and parity, oral contraceptive use, tubal ligation and family history of ovarian cancer
Merritt, M.A. [29]	2014	US	25–55	28	Cohort	Validated FFQ	88356 (155)	Highest: >675.4 Lowest: <277.7	Dairy calcium	EOC	0.86 (0.68, 1.10)	
Merritt, M.A. [27]	2013	US	52.5/52.4	NA	CC	Validated FFQ	3898 (1909)	Highest: >859.3 Lowest: <543.7	Dietary calcium	EOC	0.74 (0.62, 0.89)	Age, number of pregnancies, oral contraceptive use, tubal ligation, family history of ovarian cancer in a first degree relative, study center, study phase and total calories
								Highest: >1318.8 Lowest: <654.9	Total calcium	EOC	0.62 (0.49, 0.79)	Age, number of pregnancies, oral contraceptive use, tubal ligation, family history of ovarian cancer in a first degree relative, study center, study phase, total calories, total vitamin D and lactose
Qin, B. [30]	2016	US	57.3/54.9	NA	CC	Validated FFQ	1146 (490)	Highest: >819.6 Lowest: <362.4	Dietary calcium	EOC	0.52 (0.28, 0.98)	Age, region, and total energy intake, education, parity, oral contraceptive use, menopausal status, tubal ligation, family history of breast/ovarian cancer, daylight hours spent outdoors in summer months, pigmentation, recreational physical activity, BMI, other sugar intake excluding lactose, plus quartiles of total vitamin D, and lactose, supplemental intake of calcium
								Highest: >1233.7 Lowest: <478.6	Total calcium	EOC	0.51 (0.30, 0.86)	Age, region, and total energy intake, education, parity, oral contraceptive use, menopausal status, tubal ligation, family history of breast/ovarian cancer, daylight hours spent outdoors in summer months, pigmentation, recreational physical activity, BMI, other sugar intake excluding lactose, plus quartiles of total vitamin D, and lactose
Tzonou, A. [33]	1993	Greece	<75	NA	CC	FFQ	389 (189)	Highest: >1500 Lowest: <500	Dietary calcium	EOC	0.93 (0.38, 2.29)	Total calories
Chang, E.T. [23]	2007	US	50	8.1	Cohort	Validated FFQ	97275 (280)	Highest: >1127 Lowest: <461	Total calcium	EOC	0.90 (0.57, 1.43)	Race, total energy intake, parity, oral contraceptive use, strenuous exercise, wine consumption, and menopausal status/hormone therapy use, use of dietary supplements, excluded short-term supplement users
Bidoli, E. [31]	2001	Italy	56/57	NA	CC	Validated FFQ	3442 (1031)	NA	Dietary calcium	EOC	0.70 (0.60, 1.00)	Age, study center, year of interview, education, BMI, parity, oral contraceptive use, occupational physical activity, and energy intake
Salazar, M.E. [34]	2002	Mexico	53/54	NA	CC	Validated FFQ	713 (84)	Highest: ≥1205 Lowest: <800	Dietary calcium	EOC	0.59 (0.32, 1.10)	Age, total energy intake, number of live births, recent changes in weight, physical activity and diabetes
Kushi, L.H. [41]	1999	US	55-69	10	Cohort	FFQ	29083 (139)	Highest: >1372 Lowest: <731	Total calcium	EOC	1.66 (0.96, 2.88)	Age, total energy intake, number of live births, age at menopause, family history of ovarian cancer in a first-degree relative, hysterectomy/unilateral oophorectomy status, waist-to-hip ratio, level of physical activity, cigarette smoking, and educational level
Fairfield, K.M. [42]	2004	US	30-55	16	Cohort	Validated FFQ	80326 (301)	NA	Dietary calcium	OC	0.85 (0.36, 2.00)	Age, BMI, caffeine intake, duration of oral contraceptive use, parity, tubal ligation and smoking, energy
								NA	Total calcium	OC	1.47 (0.88, 2.47)	
Koralek, D.O. [35]	2006	US	61	NA	Cohort	Validated FFQ	31925 (146)	NA	Dietary calcium	OC	0.67 (0.43, 1.04)	Age, menopause type, parity, oral contraceptive use, and postmenopausal hormone use at baseline, energy
								NA	Total calcium	OC	0.65 (0.36, 1.16)	Total vitamin D, lactose, age, menopause type, parity, age at menarche, oral contraceptive use, and postmenopausal hormone use at baseline, energy
Chiaffarino, F. [24]	2007	Italy	56/57	NA	CC	FFQ	2904 (493)	NA	Dietary calcium	EOC	0.90 (0.89, 1.10)	Age, study center, year of interview, education, parity, oral contraceptive use, family history of ovarian and/or breast cancer in first degree relatives and energy intake
Faber, M.T. [26]	2012	Denmark	58.9/57.1	NA	CC	FFQ	2208 (554)	Highest: ≥1200 Lowest: <400	Dairy calcium	EOC	1.00 (0.68, 1.48)	Age, pregnancy, number of pregnancies, oral contraceptive use, duration of oral contraceptive use, hormone replacement therapy use, and family history of breast and/or ovarian cancer, lactose intake
Park, Y. [25]	2009	US	50–71	7	Cohort	Validated FFQ	74342 (515)	Highest: >1101 Lowest: <409	Dietary calcium	OC	1.02 (0.75, 1.37)	Energy, race/ethnicity, education, marital status, BMI, family history of cancer, vigorous physical activity, menopausal hormone therapy use, alcohol consumption, and intakes of red meat and total energy smoking, parity, oral contraceptive use, and duration of hormone replacement use, supplement calcium, and additional variables race/ethnicity, education, marital status, BMI, family history of cancer, vigorous physical activity
								Highest: >1881 Lowest: <494	Total calcium	OC	1.14 (0.85, 1.52)	Race/ethnicity, education, marital status, BMI, family history of cancer, vigorous physical activity, menopausal hormone therapy use, alcohol consumption, and intakes of red meat and total energy smoking, parity, oral contraceptive use, and duration of hormone replacement use, and additional variables race/ethnicity, education, marital status, BMI, family history of cancer, vigorous physical activity

Abbreviations: RR, relative risk; CI, confidence interval; US, United States; Total calcium, dietary plus supplemental calcium; CC, case-control study; Cohort, cohort study; EOC, epithelial ovarian cancer; OC, ovarian cancer; BMI, body mass index; FFQ, food frequency questionnaire; NA, not available.

**Table 2:** Summary risk estimates of the association between the intake of calcium and the risk of ovarian cancer.

Exposure	Outcome	Subgroup	No. of Studies	Pooled RR (95% CI)	*I*^2^ (%)	*P*_heterogeneity_
Dietary calcium	OC	All studies	13	0.80 (0.72, 0.89)	32.8	0.12
		Cohort	5	0.86 (0.74, 0.99)	0	0.614
		Case-control	8	0.75 (0.64, 0.89)	53.3	0.036
		North America	9	0.76 (0.66, 0.87)	26.4	0.209
		Europe	4	0.86 (0.75, 0.99)	18.9	0.296
		Validated FFQ	10	0.75 (0.67, 0.85)	20.5	0.254
		FFQ	3	0.91 (0.82, 1.00)	0	0.875
		Adjustment for parity				
		Yes	9	0.79 (0.69, 0.91)	42.5	0.084
		No	4	0.77 (0.66, 0.90)	0	0.424
		Adjustment for tubal ligation				
		Yes	6	0.74 (0.64, 0.86)	20.1	0.282
		No	7	0.85 (0.75, 0.97)	21.7	0.264
Dietary calcium	EOC	All studies	10	0.78 (0.69, 0.88)	40.5	0.087
Total calcium	OC	All studies	7	0.90 (0.65, 1.24)	76.1	0.000
Dairy calcium	OC	All studies	4	0.80 (0.66, 0.98)	34.5	0.205

Abbreviations: RR, relative risk; CI, confidence interval; Total calcium, dietary plus supplemental calcium; EOC, epithelial ovarian cancer; OC, ovarian cancer.

## Supplementary Materials

The following are available online at www.mdpi.com/2072-6643/9/7/679/s1, Table S1: Quality assessment of studies included in the meta-analysis, Figure S1: Meta-analysis of the association between dietary plus supplemental calcium intake and ovarian cancer risk, Figure S2: Meta-analysis of the association between dairy calcium intake and ovarian cancer risk. Figure S3: Influence analysis of an individual study on the pooled estimate for studies on the association between dietary calcium intake and ovarian cancer risk, Figure S4: Influence analysis of an individual study on the pooled estimate for studies on the association between dietary plus supplemental calcium intake and ovarian cancer risk.

## Abbreviations

*RR*	relative risk
*CI*	confidence interval
REM	random effect model
EOC	epithelial ovarian cancer
FFQs	food frequency questionnaires
PTH	parathyroid hormone
IGF-1	insulin-like growth factor-1
US	United States
