# Supplementary material for: Calcium Intake and the Risk of Ovarian Cancer: A Meta-Analysis
Source: Nutrients. 2017 Jun 30;9(7):679. doi: 10.3390/nu9070679 (PMC5537794; doi:10.3390/nu9070679)
Supplement: Supplementary file 1 [file nutrients-09-00679-s001.zip › nutrients-193945-supplementary.pdf]

Table S1. Quality assessment of studies included in the meta-analysis

First author (year)	Selection	Comparability	Outcome/exposure	Overall quality
Goodman M.T. (2002)	****	**	***	9
Merritt, M. A. (2013)	****	**	**	8
Qin, B. (2016)	****	**	***	9
Tzonou, A. (1993)	***	**	***	8
Bidoli, E. (2001)	***	**	***	8
Salazar, M. E. (2002)	***	**	**	7
Chiaffarino, F. (2007)	***	**	**	7
Faber, M. T. (2012)	****	**	**	8
Merritt, M. A. (2014)	***	*	***	7
Chang, E. T. (2007)	***	*	***	7
Kushi, L. H. (1999)	****	**	***	9
Koralek, D. O. (2006)	****	**	***	9
Park, Y. (2009)	****	*	**	7
Fairfield, K. M. (2004)	***	**	**	7

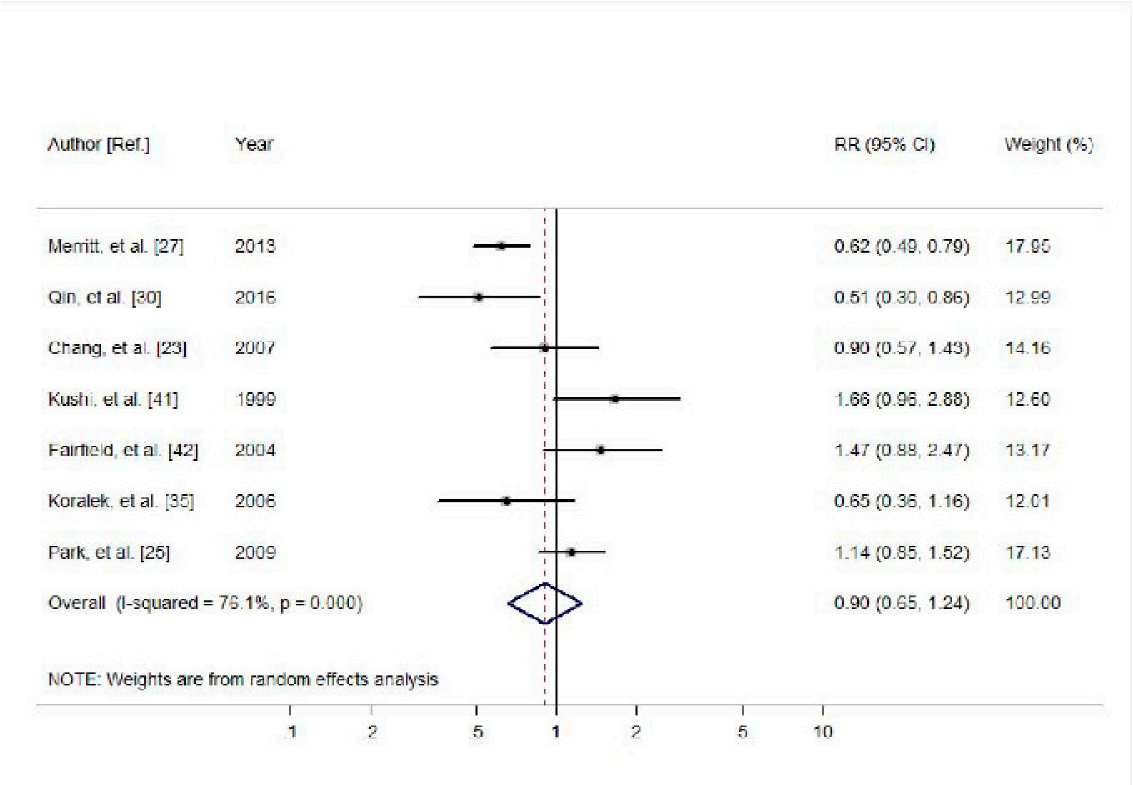

Figure S1. Meta-analysis of the association between dietary plus supplemental calcium intake and ovarian cancer risk.

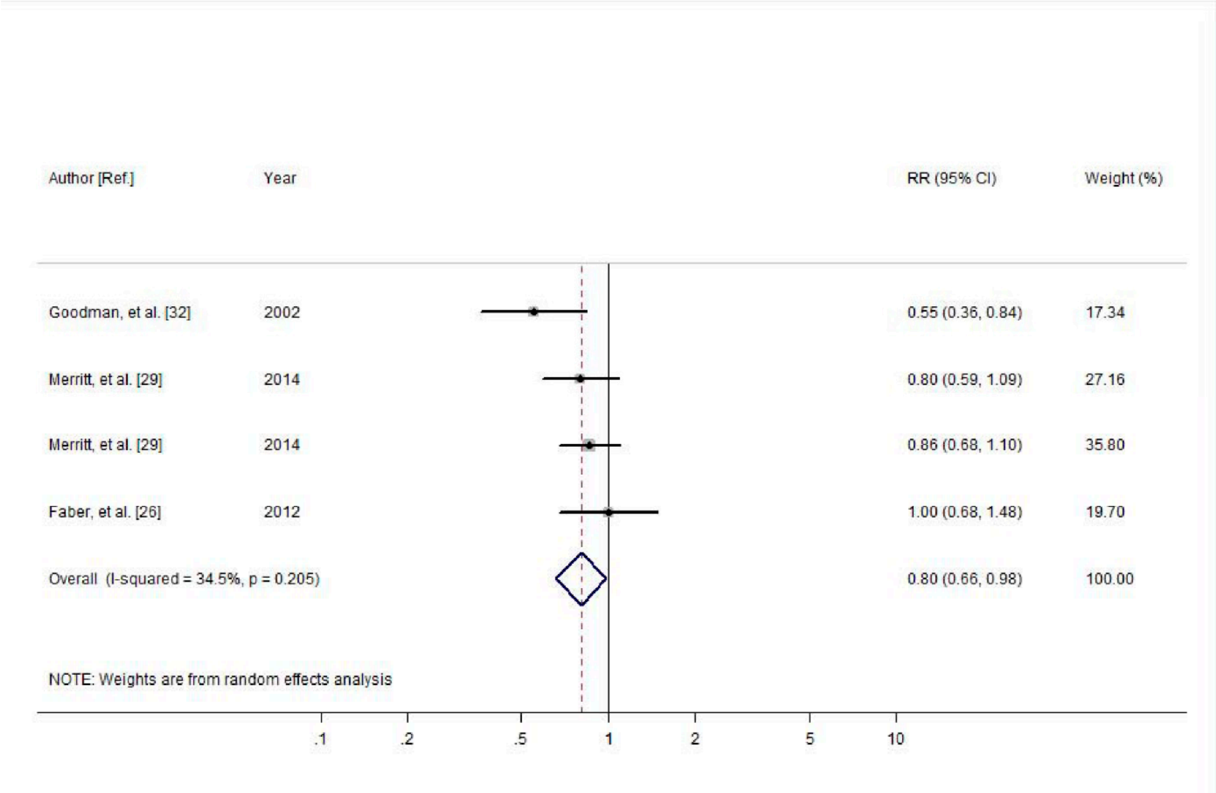

Figure S2. Meta-analysis of the association between dairy calcium intake and ovarian cancer risk.

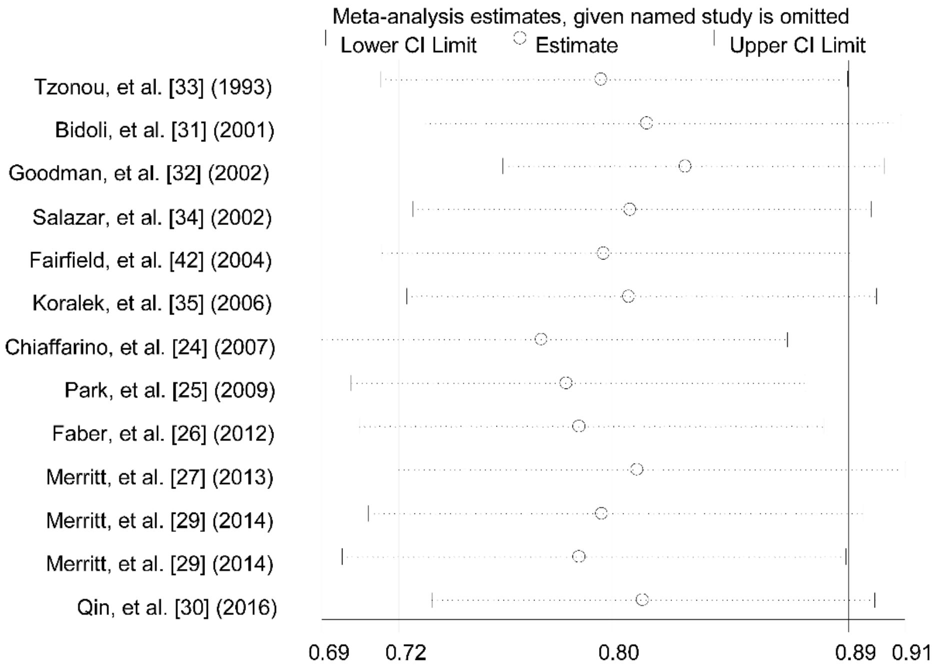

Figure S3. Influence analysis of an individual study on the pooled estimate for studies on the association between dietary calcium intake and ovarian cancer risk.

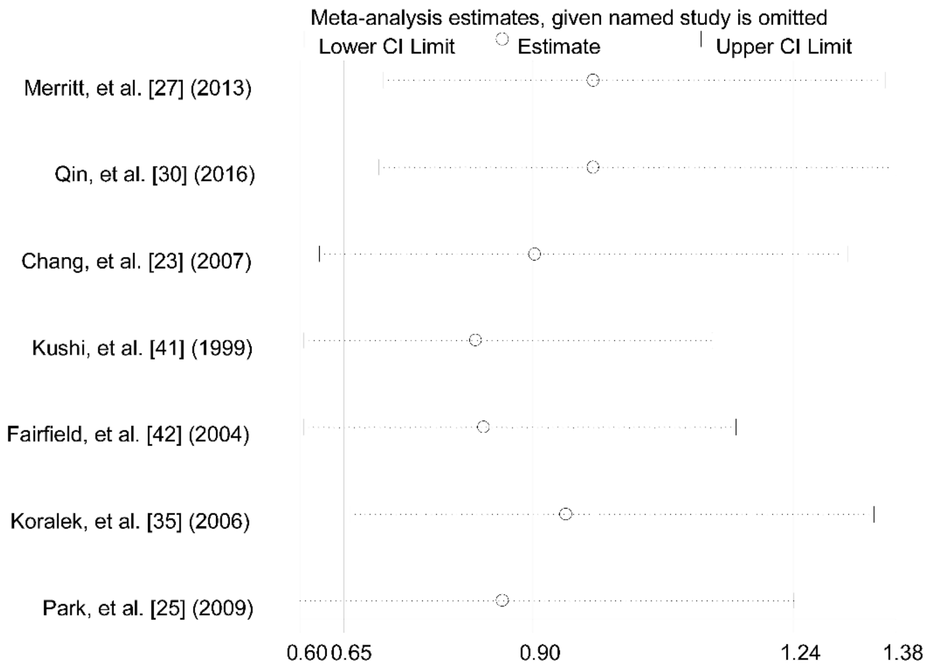

Figure S4. Influence analysis of an individual study on the pooled estimate for studies on the association between dietary plus supplemental calcium intake and ovarian cancer risk.
